# Ocular surface involvement and histopathologic changes in the acute stage of Stevens-Johnson syndrome and toxic epidermal necrolysis: a cross-sectional study

**DOI:** 10.1186/s12886-023-03052-7

**Published:** 2023-07-03

**Authors:** Yingyi Liu, Jianing Feng, Yuerong Ren, Wen Shi, Huanmin Kang, Yingqian Peng, Yixin Tan, Ruifang Wu, Guiying Zhang, Yan He

**Affiliations:** 1 Department of Ophthalmology, The Second Xiangya Hospital, Central South University, Changsha, Hunan China; 2 Hunan Clinical Research Center of Ophthalmic Disease, Changsha, Hunan China; 3 Xi’an People’s Hospital (Xi’an Fourth Hospital), Shaanxi Eye Hospital, Northwest University Affiliated People’s Hospital, Xi’an, Shaanxi Province China; 4 Department of Dermatology, The Second Xiangya Hospital, Central South University, Changsha, Hunan China

**Keywords:** Stevens-Johnson syndrome, Toxic epidermal necrolysis, Ocular surface, Conjunctival impression cytology, Tear cytokine

## Abstract

**Background:**

Stevens-Johnson syndrome (SJS) and toxic epidermal necrolysis (TEN) are rare and extremely serious drug-induced dermatological disorders. The ocular surface condition at the early stage has been little studied and should contribute to novel perspectives in early and effective topical therapy of these diseases. The objectives of the study were to evaluate the acute phase of ocular surface involvement and histopathologic changes in patients with acute SJS/TEN.

**Methods:**

Ten patients with acute phase of SJS/TEN onset and eleven age- and sex-matched healthy volunteers were recruited. Ocular surface symptoms and signs, conjunctival impression cytology, and tear multi-cytokine were assessed.

**Results:**

Ocular surface objective signs were normal at the acute stage of SJS/TEN, while most patients have abnormal ocular surface subjective symptoms and meibomian gland secretion. Conjunctival impression cytology showed a significant decrease in goblet cell density and severe ocular surface squamous metaplasia in acute SJS/TEN patients. Tear multi-cytokine analysis showed all 21 pro- and anti-inflammatory cytokines all sharply elevated. Goblet cell density was significantly negatively correlated with tear C-X3-C motif chemokine ligand 1 (CX3CL1) and interleukin 13.

**Conclusions:**

Severe pathologic squamous metaplasia and inflammation onset in the ocular surface at the acute stage of the SJS/TEN, even if the ocular surface condition seemed basically normal with adequate systemic immunosuppressant and general supportive treatment. Early topical anti-inflammatory therapy should be carried out actively.

**Supplementary Information:**

The online version contains supplementary material available at 10.1186/s12886-023-03052-7.

## Background

Stevens-Johnson syndrome (SJS) and its much more severe form, toxic epidermal necrolysis (TEN), are primarily drug-induced life-threatening immunologic dermatological disorders characterized by a sudden high fever, necrosis and detachment of skin as well as mucous membranes of the whole body [1, 2]. Although SJS/TEN are rare diseases, with an estimated annual incidence of 0.4 to 7 cases per million persons, the mortality rates of individuals with these diseases are very high, which was reported to be 1–5% for SJS and 25–40% for TEN [1].

Acute ocular involvement is very common in SJS/TEN patients with an incidence of 60–100%, it ranges from simple conjunctival hyperemia to almost complete ocular surface epithelium falling off [3, 4]. However, since the severity of ocular involvement and skin lesions are not always parallel at onset, eye conditions are easily overlooked. Early diagnosis and treatment for ophthalmic diseases in the acute phase of SJS/TEN are extremely critical but lack study [5]. Conjunctival impression cytology is a noninvasive technique that samples the conjunctival exfoliated cells and analyses them through periodic acid-Schiff (PAS) staining and immunofluorescence staining. This helps us understand the condition of ocular surface damage from a histopathologic point of view [6]. However, the majority of research has focused on ocular surface pathological changes in the chronic phase of SJS/TEN, with fewer investigations in the acute phase.

The pathogenesis of SJS/TEN is not completely clear; it is considered to be a T-cell mediated, delayed hypersensitivity activity, and cytokines play an important role in the development and progression of these diseases [7, 8]. The dynamics of multiple cytokines in the different stages of SJS/TEN were observed in epidermal lesions, blister fluid, serum, corneal epithelium, and tears [9–11]. As a noninvasive method, tear multi-cytokine analysis helps make an accurate and quantitative assessment of ocular surface inflammation. Several studies have focused on the pathophysiological mechanism of ocular surface inflammation in chronic SJS/TEN patients through tear cytokine detection [11–14]. However, the study of tear cytokine analysis for patients with acute SJS/TEN is lacking, except for one case report that monocyte chemoattractant protein-1 (MCP-1), interleukin-6 (IL-6), and interleukin-8 (IL-8) were rapidly increased in an SJS patient’s tears at the acute stage [15].

This study aims to investigate ocular subjective symptoms, objective signs, conjunctival impression cytology, and tear multi-cytokine analysis of patients with acute SJS/TEN, which may help clarify the underlying ocular pathophysiological mechanism in the acute phase and enhance the early management of these diseases.

## Materials and methods

### Study design

A cross-sectional study.

### Subjects

Our study followed the principles of the Declaration of Helsinki and was approved by the Ethics Committee of The Second Xiangya Hospital of Central South University (Ethics code: LYF2021028). All participants provided written informed consent. Ten cases aged 16 years or older with acute SJS (8 patients, 16 eyes) and TEN (2 patients, 4 eyes) were recruited from the in-patient dermatology ward of The Second Xiangya Hospital from March 2021 to September 2022. Those who had a history of intraocular surgery or ocular laser treatment, or eye infection within the past 3 months were excluded from the study. Eleven age- and sex-matched healthy volunteers were also enrolled.

The diagnosis of acute SJS/TEN by a dermatologist was based on (1) Significant acute prodromal symptoms. (2) Extensive and severe mucocutaneous diseases with rash, in which at least two mucosal sites were involved. SJS involves less than 10% of the body surface area, and TEN involves more than 30% of the body surface. (3) The first 2 months after the onset of clinical symptoms and without any ocular cicatricial findings such as symblepharon, conjunctivalization, and corneal neovascularization was defined as the acute phase [16–18].

### Ocular surface disease index (OSDI)

The OSDI questionnaire is a recognized standard to evaluate subjective symptoms of dry eye diseases that contained 12 questions and covered three subscales (vision-related function, ocular symptoms, and environmental triggers). The total OSDI scores = [(sum of severity for all questions answered) × 100] / [(total number of questions answered) × 4], which ranged from 0 to 100. Based on the OSDI scores, the severity of dry eye diseases was classified into 2 grades: 0 = normal (scores ≤ 22) and 1 = abnormal (scores > 22) [19].

### Ocular surface assessments

#### Dry eye diseases assessment

##### Corneal and conjunctival fluorescent staining (FL) grade

To assess the integrity of corneal and conjunctival epithelium. Fluorescein sodium was used to stain the cornea, and whether the cornea was stained under cobalt blue light was observed. The FL grade is based on the Oxford grading scheme (grades 0–5). Normal FL grade = 0 [20].

##### Schirmer I test

To assess the reflex tear secretion. A tear secretion test strip was placed in the outer one-third of the lower conjunctival sac without topical anesthesia. All participants were asked to close both eyes for 5 min, and the values on the test strip were measured. If the difference between the two eyes was significant, a second test was performed. Normal Schirmer I test > 5 mm/5 min [21].

##### Tear break-up time (TBUT)

To assess the tear film stability. Fluorescein sodium was instilled into the inferior conjunctival sac, all the participants were instructed to blink 3 times to distribute the fluorescein, and open their eyes naturally after the last complete blink. The duration required for the first dark spot on the cornea was determined. Normal TBUT > 10 s [21]. For statistical use, we defined normal (FL grade = 0, Schirmer I test > 5 mm/5 min, TBUT > 10 s) as 0 and abnormal (FL grade > 0, Schirmer I test ≤ 5 mm/5 min, TBUT ≤ 10 s) as 1.

#### Lid parallel conjunctival folds (LIPCOF) (grades 0–3)

LIPCOF was reported as a test with a high predictive ability for dry eye diseases and conjunctivochalasis [22, 23]. The area perpendicular to the nasal and temporal bulbar conjunctiva in the lower lid was evaluated and scored on 0–3 grades (0 = no conjunctival folds, 1 = one permanent and clear parallel fold, 2 = two permanent and clear parallel folds, and 3 = more than two permanent and clear parallel folds) [24].

#### Meibomian gland secretions (grades 0–3)

Meibomian gland dysfunction is characterized by obstruction of the meibomian gland ducts and/or abnormality in the glandular secretion, which is the main cause of evaporative dry eye. The upper eyelids of participants were moved and gently squeezed, and secretions of the upper meibomian glands were observed. Clear fluid was defined as grade 0, cloudy fluid was defined as grade 1, granular and cloudy fluid was defined as grade 2, and inspissated, toothpaste-like secretions were defined as grade 3 [25].

### Conjunctival impression cytology and goblet cell evaluation

We collected cells from the supratemporal and nasal bulbar conjunctiva from all participants using a 10 mm diameter semicircular and sterile cellulose acetate membrane (Advantec, Tokyo, Japan) after instilling oxybuprocaine hydrochloride eye drops for topical anesthetic. The collection area is approximately 39.25 mm^2^. The specimens were fixed in 95% ethanol or 4% paraformaldehyde (PFA), followed by PAS staining or MUC5AC immunofluorescence staining.

#### PAS staining

PAS staining was carried out by using a glycogen staining kit (G1360, Solarbio, Beijing, China). The specimens fixed with 95% ethanol were removed and washed with distilled water. Subsequently, the ethylene glycol present in the mucin of goblet cells was oxidized by periodic acid into dialdehydes, which reacted with Schiff reagent and generated a purple-magenta color. Finally, specimens were dehydrated in gradient alcohol, cleared in xylene, sealed with neutral balsam, and air-dried until observation [26].

#### MUC5AC immunofluorescence staining

MUC5AC is the most important component of the mucin layer of the tear film. The 4% PFA-fixed specimens were washed with 0.1% Tween-20 in phosphate buffered saline (PBS) and then blocked with 10% donkey serum (Absin Bioscience, Shanghai, China) and 1% bovine serum albumin (BSA) in PBS for 1 h at room temperature. Later, specimens were incubated at 4 °C overnight with 1% BSA and 0.3% Triton X-100 in PBS containing the primary antibody anti-MUC5AC (1:200; ab3649, Abcam, Cambridge, UK). Afterward, specimens were washed with 0.1% Tween-20 in PBS and then incubated with Alexa Fluor® 594-AffiniPure Donkey Anti-Mouse IgG secondary antibody (1:100; 715–585-150, Jackson Immune Research, Pennsylvania, US) for 2 h at room temperature. Specimens were then washed and mounted with the 4',6-diamino-2-phenylindole (DAPI) histology mounting medium (Sigma Aldrich, St. Louis, Missouri, USA) and were stored at 4 °C until observation.

All specimens were collected using an Invitrogen™ EVOS™ M7000 fully automatic live-cell fluorescence microscopy imaging system (Thermo Fisher Scientific, Massachusetts, U.S.), and all images were analyzed by ImageJ software (National Institutes of Health, Maryland, USA). Five non-overlapping regions were randomly captured from each specimen with a × 20 objective lens. The data are presented as the average goblet cell density (cells/mm^2^) and average MUC5AC^+^ goblet cell density (cells/mm^2^) of both eyes. Nelson’s grading system was used to evaluate the degree of ocular surface squamous metaplasia. Higher scores indicate greater ocular surface squamous metaplasia [27].

### Tear multi-cytokine analysis

#### Tear sample collection

Ten µl 0.9% normal saline was instilled in the participants’ inferior conjunctival sac. We placed the tip of disposable microcapillary fluid collectors (Seinda, Guangdong, China) in the outer one-third of the lower conjunctival sac, and then tear entered the collectors by the siphoning effect. A 2.2 µl tear sample was collected per tube 3 times per eye at 10-min intervals. Subsequently, the tear samples were transferred to sterile 200 µl centrifuge tubes and immediately stored at -80 ℃ for further use.

#### Tear cytokine detection

A Milliplex Map Human High Sensitivity T-Cell Panel-Immunology Multiplex Assay (Millipore, Billerica, MA, USA) was used to estimate the concentration of tear cytokines following the manufacturer's procedure, including granulocyte–macrophage colony stimulating factor (GM-CSF), C-X-C motif chemokine ligand 11 (CXCL11), C-X3-C motif chemokine ligand 1 (CX3CL1), interferon gamma (IFN-γ), tumor necrosis factor alpha (TNF-ɑ), IL-1β, IL-2, IL-4, IL-5, IL-6, IL-7, IL-8, IL-10, IL-12, IL-13, IL-17A, IL-21, IL-23, C–C motif chemokine ligand 3 (CCL3), C–C motif chemokine ligand 4 (CCL4), and C–C motif chemokine ligand 20 (CCL20). Tears were all from the right eye. Briefly, 2 µl of tear sample was removed and diluted with 0.9% normal saline, and then transferred to the corresponding sample hole. The positive control group was a standard with a gradient concentration, and the negative controls only contained an equivalent amount of solvent. Microbeads and antibodies were added to each well for a period of incubation, followed by a period of washing. Then, tear cytokines were detected by MAGPIX liquid-phase chip detector (Luminex, Austin, TX, USA) with xPONENT® software (Luminex, Austin, TX, USA). The tear cytokine concentrations of each sample were calculated by standard curve fitting.

### Statistical analysis

Measurement data were expressed as the mean ± standard deviation (SD), categorical data, and ranked data were expressed as frequencies and percentages. The Shapiro–Wilk test was used to check the normality of measurement data. The independent-sample t-test was used for normally distributed measurement data, and the Mann–Whitney U test was used for nonnormally distributed measurement data and ranked data. Chi-squared test was performed for categorical data. Correlation analysis was performed using Pearson's test for normal distribution or Spearman's test for nonnormal distribution. SPSS 20.0 software was used for statistical analysis, with a *P* value < 0.05 accepted as statistically significant.

## Results

### Basic patient information

**Fig. 1:**
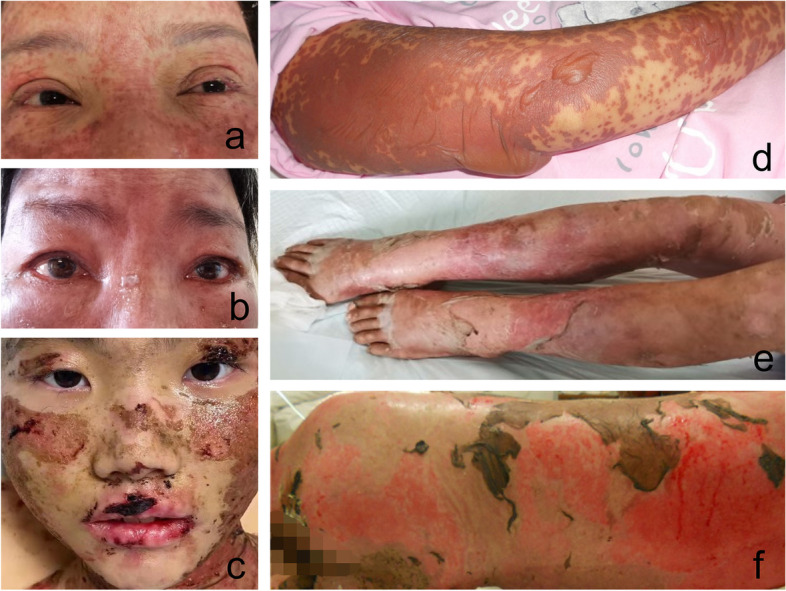
Representative images of acute SJS/TEN patients. **a** Ocular surface image of a patient with acute SJS (number 7 patient in Table 1). Diffuse erythema around both eyelids, conjunctival hyperemia, and edema of both upper eyelids. The image was taken on the fourth day after the onset of clinical symptoms. **b** Ocular surface image of a patient with acute TEN (number 9 patient in Table 1). Conjunctival hyperemia and edema of bulbar conjunctiva in both eyes. The image was taken on the fifth day after the onset of clinical symptoms. **c** Facial image of a patient with acute TEN (number 10 patient in Table 1). Facial peeling and lip erosion. The image was taken on the eleventh day after the onset of clinical symptoms. **d** Arm image of a patient with acute SJS (number 8 patient in Table 1). Diffuse erythema, partial epidermolysis, flaccid vesicles, and bullae (positive Nissl's sign) on the arm. The image was taken on the tenth day after the onset of clinical symptoms. **e** Leg image of a patient with acute TEN (number 9 patient in Table 1). Skin sloughing on both legs. The image was taken on the fifth day after the onset of clinical symptoms. **f** Back image of a patient with acute TEN (number 10 patient in Table 1). Extensive peeling of the skin on the back. The image was taken on the eleventh day after the onset of clinical symptoms

In this study, we recruited 10 patients (8 SJS and 2 TEN), with a mean age of 52.9 ± 14.6 years. Among them, six patients (60%) were women, and four (40%) were men. They all had an average general disease course of 7.8 ± 2.9 days (range from 4–13 days, all within 2 months, in their acute phase). Four patients (40%) had fever at admission, of which two (20%) had a body temperature equal to or greater than 39 °C. Seven patients (70%) had a clear history of drug exposure, of which four cases (40%) were induced by carbamazepine. Six cases (60%) had more than five damaged skin and mucosal sites except for the eyes, and two of them (20%) had diffuse skin lesions all over the body. Nine patients (90%) were given intravenous immunoglobulin, and all were given intravenous methylprednisolone. There were seven patients (70%) with a history of systemic diseases and three patients (30%) with a history of eye diseases. Moreover, one patient (10%) died due to aggravation of the poor general condition, and the other nine patients (90%) had incomplete clinical recovery (Table 1). Representative images of patients with acute SJS/TEN are shown in Fig. 1.

**Table 1:** Basic information on acute SJS/TEN patients

Number	Diagnosis	Age (year)	Gender	Duration from the onset of disease (day)	Body temperature at admission (°C)	Causative medications	Other damaged skin and mucosal sites	Systemic therapy	History of systemic diseases	History of eye diseases	Disease outcome
1	SJS	63	F	8	37.0	Carbamazepine	Trunk, limbs, palms, toes, back, face, oral mucosa, lips, and perineum	MePr and γ-globulin (I.V)	Hypertension and coronary heart disease	N	IR
2	SJS	50	F	4	39.2	Carbamazepine	Trunk, limbs, face, neck, oral mucosa, and perineum	MePr and γ-globulin (I.V)	Gastric ulcer and fatty liver	N	IR
3	SJS	65	M	13	37.1	Carbamazepine	Diffuse erythema present all over the body	MePr and γ-globulin (I.V)	Drug-induced liver injury and lung infection	Pterygium (OU)	IR
4	SJS	41	M	9	38.2	Carbamazepine	Trunk, limbs, face	MePr and γ-globulin (I.V)	N	N	IR
5	SJS	55	M	8	37.1	Traditional Chinese medicine, amoxicillin, and ibuprofen	Trunk, limbs, neck	MePr and γ-globulin (I.V)	Type 2 diabetes, diabetic nephropathy, and diabetic peripheral neuropathy	Diabetic retinopathy (OU)	Death
6	SJS	61	M	6	37.0	Eperisone hydrochloride	Trunk, limbs, lips, and face	MePr (I.V)	N	N	IR
7	SJS	59	F	4	39.0	Allopurinol	Trunk, limbs, face, neck, oral mucosa, sole, and perineum	MePr and γ-globulin (I.V)	Type 2 diabetes, rheumatoid arthritis, and hyperuricemia	N	IR
8	SJS	50	F	10	37.1	Unknow	Trunk, limbs, oral mucosa, lips, and perineum	MePr and γ-globulin (I.V)	Type 2 diabetes, diabetic peripheral neuropathy, diabetic ketoacidosis, lung adenocarcinoma, and fatty liver	N	IR
9	TEN	67	F	5	38.5	Unknow	Scalp, back, limbs, face	MePr and γ-globulin (I.V)	Cerebral infarction and psoriasis	Acute retinal necrosis (OU), retinal artery occlusion (OU), and retinal vasculitis (OU)	IR
10	TEN	18	F	11	37.1	Unknow	Diffuse erythema and exfoliation are present all over the body	MePr and γ-globulin (I.V)	N	N	IR

*SJS* Stevens–Johnson syndrome, *TEN* Toxic epidermal necrolysis, *F* Female, *M* Male, *MePr* Methylprednisolone, *γ-globulin* gamma globulin, *I.V* intravenous injection, *N* None, *OU* Both eyes, *IR* Incomplete recovery

### Ocular surface conditions in acute SJS/TEN

We recruited 11 age- and sex-matched healthy volunteers (mean age: 55.4 ± 14.1 years), which were composed of 8 women and 3 men without systemic or ocular immune-related diseases. For all participants, we evaluated the ocular surface conditions, including subjective symptoms of dry eye diseases reflected by OSDI scores, and objective signs of dry eye diseases reflected by corneal and conjunctival FL, Schirmer I test, and TBUT. LIPCOF and meibomian gland secretions were also assessed. The results are shown in Table 2. All acute SJS/TEN patients (100%) had abnormal OSDI scores (> 22), which reflected that the subjective symptoms of dry eye diseases were serious, but there was no statistically significant difference between acute SJS/TEN patients and healthy volunteers (*P* value > 0.05). Concerning the objective signs of dry eye diseases, thirteen out of twenty eyes (65%) had intact corneal and conjunctival epithelium (FL grade = 0), sixteen out of twenty eyes (80%) had normal reflex tear secretion (Schirmer I test > 5 mm/5 min), and ten out of eighteen eyes (55.6%) had normal tear film stability (TBUT > 10 s). The differences in FL grade, Schirmer I test, and TBUT between the acute SJS/TEN patients and healthy volunteers were not statistically significant (*P* value > 0.05). In addition, twelve out of twenty eyes (60%) had no conjunctival fold (LIPCOF = 0), and the differences in LIPCOF between the acute SJS/TEN patients and healthy volunteers were not statistically significant (*P* value > 0.05). However, the grading of meibomian gland secretions was significantly higher in acute SJS/TEN patients than in healthy volunteers (*P* value < 0.05), and eighteen out of twenty eyes (90%) acute SJS/TEN patients had abnormal meibomian gland secretions (> 0).

**Table 2:** Ocular surface conditions and conjunctival impression cytology of acute SJS/TEN patients

Number	Diagnosis	OSDI scores (0–100)	Ocular surface assessments										Goblet cell density (cells/mm^2^)	MUC5AC^+^ goblet cell density (cells/mm^2^)	Nelson’s grades (0–3)
			**FL grades (0–5)**		**Schirmer I test (mm)**		**TBUT (s)**		**LIPCOF grades (0–3)**		**Meibomian gland secretions grades (0–3)**				
			**OD**	**OS**	**OD**	**OS**	**OD**	**OS**	**OD**	**OS**	**OD**	**OS**			
1	SJS	72.9	4	3	8	12	> 10	> 10	1	2	2	2	NA	59	NA
2	SJS	22.9	0	0	> 10	> 10	> 10	> 10	0	0	1	1	NA	NA	NA
3	SJS	31.3	0	0	2	4	> 10	> 10	0	0	2	2	36.25	35.98	3
4	SJS	NA	0	0	> 10	> 10	> 10	> 10	0	0	0	0	NA	NA	NA
5	SJS	50	3	3	6	8	8	6	1	2	1	1	82.54	60	3
6	SJS	22.9	2	0	4	8	5	8	0	0	3	3	93.47	5.83	3
7	SJS	58.3	0	0	5	8	10	10	3	3	2	2	87	80	3
8	SJS	56.3	1	1	8	9	NA	NA	0	0	3	2	97.5	93.33	3
9	TEN	41.6	0	0	6	6	10	8	0	0	3	2	18.3	16.63	3
10	TEN	NA	0	0	14	24	> 10	> 10	2	3	2	2	NA	NA	NA

*SJS* Stevens-Johnson syndrome, *TEN* Toxic epidermal necrolysis, *NA* Not available, *OD* Right eye, *OS* Left eye, *OSDI* Ocular surface disease index, *FL* Fluorescent staining; *TBUT* Tear break-up time, *LIPCOF* Lid parallel conjunctival folds

### Conjunctival impression cytology in acute SJS/TEN

**Fig. 2:**
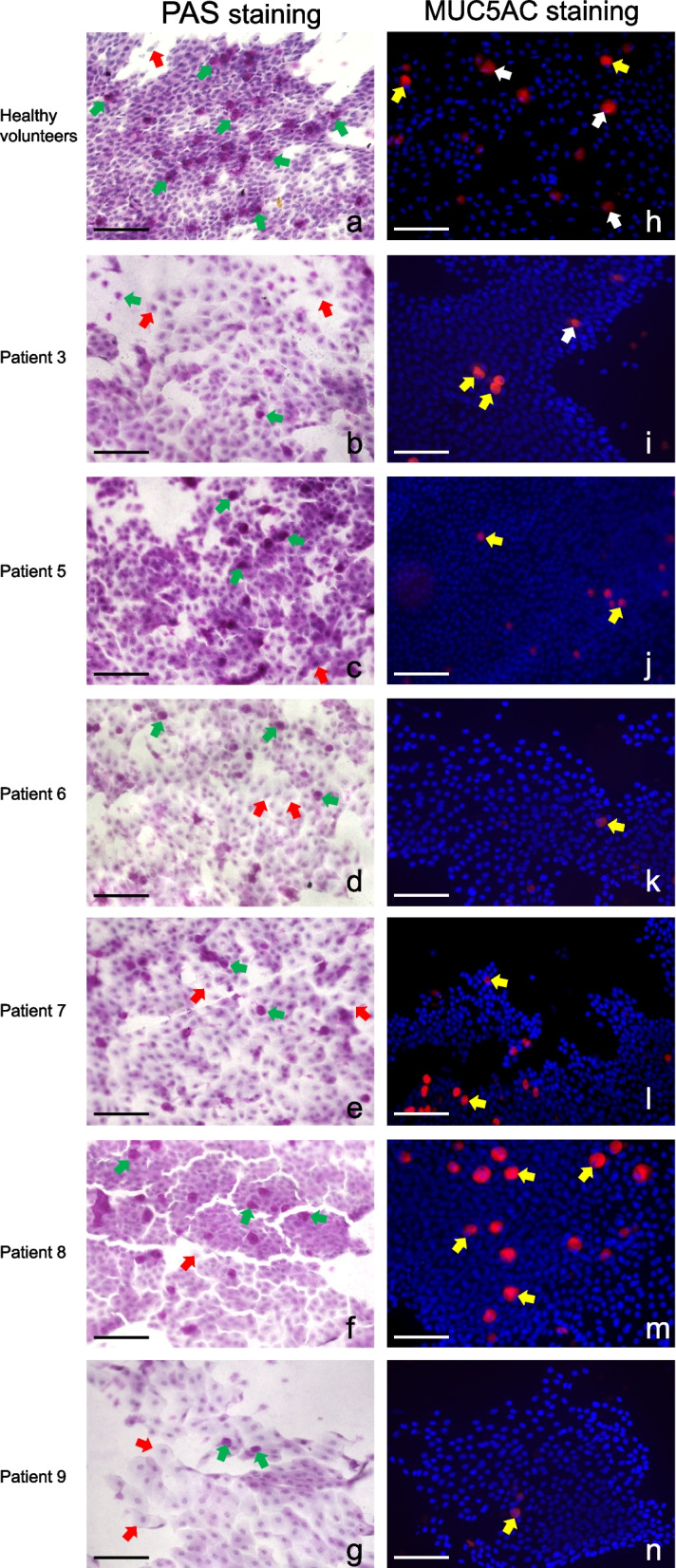
Representative images of PAS staining and MUC5AC staining in acute SJS/TEN patients and healthy volunteers (Scale bars = 100 μm). **a** PAS staining of conjunctival impression cytology in a healthy volunteer (× 200, Nelson’s grade 0). Goblet cells (green arrow) are abundant. Nonsecretory epithelial cells (red arrow) are small and round and have large nuclei with a nuclear-cytoplasmic ratio = 1:2. **b**-**g** PAS staining of conjunctival impression cytology in patients with acute SJS/TEN (× 200, Nelson’s grade 3). Nonsecretory epithelial cells (red arrow) are slightly separated and have small and pyknotic nuclei with decreased nuclear-cytoplasmic ratio, and goblet cells (green arrow) are significantly reduced. **h** MUC5AC staining of conjunctival impression cytology in a healthy volunteer (× 200). MUC5AC^+^ goblet cells include a non-degranulated pattern with complete cell boundaries and intracellularly packaged mucins (yellow arrow) and a degranulated pattern with blurred cell boundaries and scattered mucin particles (white arrow). **i**-**n** MUC5AC staining of conjunctival impression cytology in patients with acute SJS/TEN (× 200). MUC5AC.^+^ goblet cells were almost all in the form of non-degranulated (yellow arrow)

We used conjunctival impression cytology, a simple and noninvasive technique, to assess the ocular surface health of six acute SJS/TEN patients and eleven healthy volunteers. Here, PAS staining and Nelson’s grading system [27] were used to define ocular surface squamous metaplasia, which focuses on the goblet cell density, the nucleus-cytoplasmic (N: C) ratio, and the shape and cell size of nonsecretory epithelial cells. The average conjunctival goblet cell density in acute SJS/TEN patients (69.18 ± 33.35 cells/mm^2^) was less than half of that in healthy volunteers (150.18 ± 84.17 cells/mm2, *P* value < 0.05). Meanwhile, Nelson’s grades of acute SJS/TEN patients were all at the most severe level, and the difference was statistically significant compared to healthy volunteers (*P* value < 0.05, Table 2), which means that acute SJS/TEN patients sharply developed severe ocular surface squamous metaplasia at their very early period of the diseases. The average MUC5AC^+^ goblet cell density was lower in acute SJS/TEN patients (50.11 ± 32.2 cells/mm^2^) than in healthy volunteers (72.33 ± 30.56 cells/mm2), but the difference was not statistically significant (*P* value > 0.05, Table 2). Meanwhile, almost all of the MUC5AC^+^ goblet cells on the ocular surface in acute SJS/TEN patients follow a low-secretory pattern (mainly a non-degranulated pattern with complete cell boundaries and intracellularly packaged MUC5AC mucins). Representative images of PAS staining and MUC5AC staining of conjunctival impression cytology in acute SJS/TEN patients and healthy volunteers are shown in Fig. 2.

### Tear cytokine storm in acute SJS/TEN

**Fig. 3:**
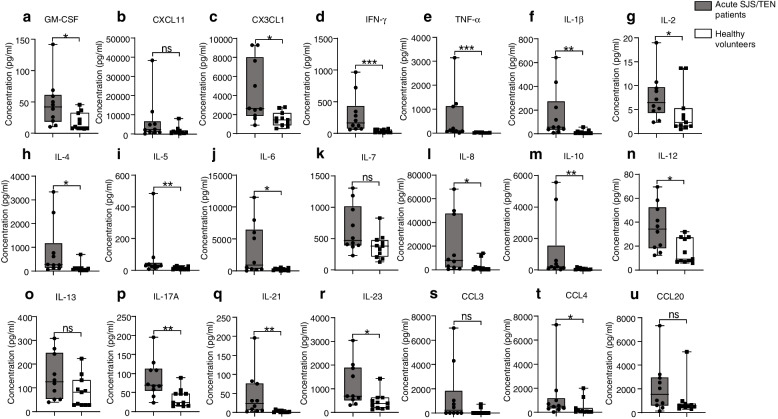
The box plot of tear cytokines level in acute SJS/TEN patients and healthy volunteers. *P value < 0.05; **P value < 0.01; ***P value < 0.001; ns: not significant; GM-CSF: granulocyte–macrophage colony stimulating factor; CXCL11: C-X-C motif chemokine ligand 11; CX3CL1: C-X3-C motif chemokine ligand 1; IFN-γ: interferon gamma; TNF-ɑ: tumor necrosis factor alpha; IL: interleukin; CCL: C–C motif chemokine ligand

As shown in Fig. 3, all tear cytokines we tested tended to increase in the tears of acute SJS/TEN patients over healthy volunteers with the Luminex assay. We observed significant upregulation of GM-CSF, CX3CL1, IFN-γ, TNF-ɑ, IL-1β, IL-2, IL-4, IL-5, IL-6, IL-8, IL-10, IL-12, IL-17A, IL-21, IL-23, and CCL4 in the tears of SJS/TEN patients at their acute stage compared to those in healthy volunteers (*P* value < 0.05). The levels of TNF-ɑ and IL-10 were 32-fold and 20-fold increased, respectively. Although the levels of CXCL11, IL-7, IL-13, CCL3, and CCL20 were also increased in the acute SJS/TEN patients in comparison to healthy volunteers, the differences were not statistically significant (*P* value > 0.05). Besides, the levels of 21 tear cytokines in male acute SJS/TEN patients were all not significantly elevated compared with male healthy volunteers (*P* value > 0.05), while GM-CSF, CXCL11, IFN-γ, TNF-α, IL-1β, IL-5, IL-6, IL-8, IL-10, IL-17A, IL-21, IL-23, CCL4, and CCL20 were significantly elevated in female acute SJS/TEN patients compared with female healthy volunteers, as shown in supplementary Fig. 1.

### Factors associated with tear cytokines in acute SJS/TEN patients

**Fig. 4:**
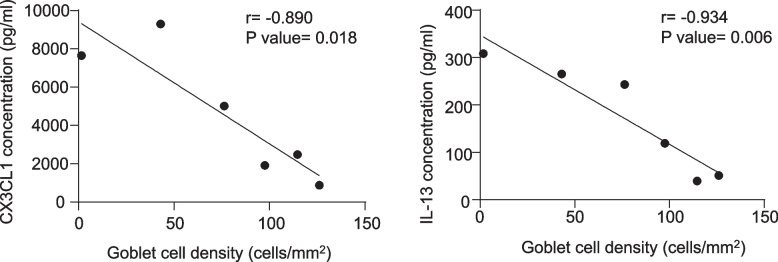
Correlation analysis between goblet cell density and tear cytokines in acute SJS/TEN patients. r: Spearman rank correlation coefficient

To further understand the relationship between ocular surface status and tear cytokines in acute SJS/TEN patients, we conducted a correlation analysis between tear cytokines and conjunctival goblet cell density, MUC5AC^+^ goblet cell density, and meibomian gland secretions. All tear cytokines were not significantly associated with MUC5AC^+^ goblet cell density and meibomian gland secretions (*P* value > 0.05), while tear CX3CL1 (*r* = -0.890, *P* value < 0.05) and IL-13 (*r* = -0.934, *P* value < 0.01) were negatively correlated with conjunctival goblet cell density (Fig. 4).

## Discussion

Historically, SJS was first described by two American pediatricians named Stevens and Johnson in 1922 and occurred in two children characterized by persistent fever, widespread skin eruption, and serious purulent conjunctivitis [28]. TEN was first reported in 1956 by Lyell, who described four cases with exfoliative mucocutaneous resembling burns [29]. SJS/TEN is one of the rare skin diseases that constitute a true medical emergency. Acute conjunctivitis, simultaneously or followed by skin lesions accompanied by extraordinarily high fever, nails, and oral mucosa involvement, indicates the onset of SJS/TEN [18]. Even if these patients survive the severe acute illness, serious sequelae often last a lifetime. Ocular complications are considered to be the most common complication among SJS/TEN survivors [30].

As reported, at least one-third of acute ocular manifestations may develop into chronic sequelae, including severe dry eye diseases, symblephara, trichiasis, and persistent corneal epithelial defects, which have an obvious effect on the quality of SJS/TEN survivors’ life [5, 31]. However, effective treatment for chronic sequelae is lacking [32]. Therefore, increased awareness and understanding of acute ocular involvement of SJS/TEN are needed. Positive and proper management of acute ocular involvement is essential to decelerate ongoing damage and prevent late complications [5, 33]. In our study, we paid attention to the acute phase of ten SJS/TEN patients. The limitation of this study is the sample size of patients since SJS/TEN is a very rare disease. We found the ocular objective signs seemed basically normal. These findings are similar to a previous study: no or mild ocular involvement was present in one-half of patients at their acute stage [34]. It is worth noting that most acute SJS/TEN patients have abnormal meibomian gland secretions, which may eventually develop into meibomian gland dysfunction and severe evaporative dry eye. As previously reported, acute ocular involvement is a risk factor for serious meibomian gland dysfunction in chronic SJS patients [35].

Goblet cells at the ocular surface secrete mucins, maintain tear film stability and lubricate the eyes [36, 37]. In this work, we first described the morphology of conjunctival epithelial cells and goblet cells and evaluated the ability of conjunctival goblet cells to secrete MUC5AC in SJS/TEN patients in the very early stage by conjunctival impression cytology. Conjunctival necrosis and inflammation lead to the loss of goblet cells and mucin in the acute phase of SJS or TEN, which eventually leads to advanced dry eye diseases that are deeply troubling for many patients [38]. In the current study, all patients had serious ocular surface squamous metaplasia, reflected by a marked reduction in goblet cells and enlarged epithelial cells, which even occurred four days from the onset of the disease. The appearance of these ocular pathological changes was significant before the ocular signs. MUC5AC is the main functional gel-forming mucin secreted by conjunctival goblet cells into tears [36]. The ability of goblet cells to produce MUC5AC is acceptable in the acute phase, which may be due to compensatory effects. Moreover, almost all of the MUC5AC^+^ goblet cells were non-degranulated on the ocular surface in acute SJS/TEN patients, indicating a dominant pattern of MUC5AC low-secretory as the ratio of degranulated to non-degranulated MUC5AC^+^ goblet cells were a marker of secretion [39]. The secretion of MUC5AC by conjunctival goblet cells is predictable to decompensate and significantly reduced in the chronic phase of the disease, which is consistent with the reports of a significant decrease or even a complete absence in conjunctival MUC5AC expression/goblet cells in the chronic phase of SJS/TEN [38, 40].

The inflammatory cascade reaction is considered to play a significant role in the pathogenesis of dry eye diseases, which eventually leads to ocular surface damage via up-regulated expression of inflammatory cytokines [41]. This is the first study to comprehensively analyze tear cytokines in patients with acute SJS/TEN. The 21 cytokines we detected in tears can be divided into 6 categories: (1) T-helper 1 cytokines: IFN-γ, IL-2, IL-7, and IL-12 that are involved in pro-inflammatory response and cellular immunity; (2) T-helper 2 cytokines: IL-4, IL-5, and IL-13 that are involved in anti-inflammatory response and humoral immunity; (3) T-helper 17 cytokines: IL-17A, IL-21, and IL-23 that are involved in proinflammatory processes and defense against extracellular bacteria and fungi; (4) T-regulatory cytokines: IL-10 that is chiefly involved in immunosuppression; (5) Proinflammatory cytokines: GM-CSF, TNF-α, IL-1β, IL-6, and IL-8; and (6) Chemokine: CXCL11, CX3CL1, CCL3, CCL4, and CCL20 that are chiefly involved in proinflammatory response [42]. In addition to this, previous studies have shown that IL-1β, IL-6, IL-8, and IL-10 were all involved in the regulation of angiogenesis [11]. TNF-α was involved in the promotion of apoptosis, while IL-7 was involved in the inhibition of apoptosis. IL-2, IL-8, and GM-CSF were involved in promoting fibrosis, while TNF-α, IL-12, and IFN-γ involved in the prevention of fibrosis [12, 14]. In our study, tear cytokines were all sharply elevated in acute SJS/TEN patients causing ocular surface cytokine storms, and showing female bias patterns without obvious alteration in males. This may be related to sex hormones [43]. Tear cytokines including pro- and anti-inflammatory cytokines, pro- and anti-apoptotic cytokines, as well as pro- and anti- fibrosis cytokines all increased, indicating a dysregulated immune response and complete loss of ocular surface homeostasis.

Similar to a previous case report [15], our study also found that IL-6 and IL-8 in SJS/TEN patients in the acute phase were significantly upregulated. However, unlike the decreased expression of IL-10, TNF-α, and IL-12 in chronic SJS/TEN [11, 14], our research showed an increase of such cytokines in the acute disease phase. Furthermore, a mixed Th1/Th2 pattern and mixed Th17/Tregs pattern was proved on the ocular surface of SJS/TEN patients in the acute phase due to Th1, Th2, Th17, and Tregs-related cytokine were all increased. This is similar to the results of a previous immunohistochemistry study on skin biopsy tissue in patients with SJS/TEN [8]. IL-13 is mainly secreted by Th2 cells but also can be secreted by mast cells, and is frequently linked to Th2 cell-related immune responses including allergic diseases, the IgE response, and parasitic infections [44]. Chemokine CX3CL1 plays an important role in mediating many inflammatory processes and tissue injury [45]. Conjunctival goblet cells were negatively correlated with tear CX3CL1 and IL-13 in acute SJS/TEN patients, indicating the degree of ocular surface squamous metaplasia increases with ocular surface inflammation. These cytokines may be the predictive biomarkers for the progression of ocular surface involvement in acute SJS/TEN. However, an important limitation of the study is we did not examine the levels of tear antifibrotic cytokine interferon-γ-induced protein 10 (IP-10) and apoptotic-associated cytokines granulysin, which have been proposed to play an important role in the onset of SJS/TEN [10, 12].

It is worth noting that, even if the patients in our study already receive active systemic glucocorticoid and immune globulin therapy, their ocular surface still suffers from a violent cytokine storm, which represents the organs and tissues that produce tears and palpebral sebum, containing lacrimal glands, accessory lacrimal glands, meibomian glands, ocular surface epithelial cells, and conjunctival goblet cells, all experience a strong immune attack at the very early stage of diseases or even before the onset of ocular symptoms and signs. This strongly suggests that even if there are no clinical ocular symptoms or signs, topical anti-inflammatory treatment may be necessary for the very early stage of these diseases. Systemic immunosuppressant treatment plays a weak role in suppressing ocular surface inflammation [46]. More attention should be given to topical treatment, including artificial tears, autologous serum eye drops, corticosteroid eye drops, and immunosuppressant eye drops, even amniotic membrane transplantation. Amniotic membrane transplantation can effectively reduce inflammation, inhibit fibrocyte response and scar formation, and stabilize the ocular surface microenvironment [47, 48], it was highly suggested to be performed in the early stage of SJS/TEN in our study.

## Conclusions

In conclusion, our study revealed that the ocular surface of SJS/TEN patients showed severe pathological squamous metaplasia and inflammations at the early stage after skin rash onset, even in patients with slight ocular symptoms and signs. These findings suggested that patients with SJS/TEN likely require a supplemental topical anti-inflammatory therapy to stabilize the ocular micro-environment.

## Supplementary Information

**Additional file 1: Fig. S1.** The box plot of tear cytokines level between male vs female in acute SJS/TEN patients and healthy volunteers. *P value <0.05; **P value <0.01; ***P value <0.001; ns: not significant; GM-CSF: granulocyte-macrophage colony stimulating factor; CXCL11: C-X-C motif chemokine ligand 11; CX3CL1: C-X3-C motif chemokine ligand 1; IFN-γ: interferon gamma; TNF-ɑ: tumor necrosis factor alpha; IL: interleukin; CCL: C-C motif chemokine ligand.

## Data Availability

The datasets that derive conclusions of this study are available from the corresponding author upon reasonable request.

## Abbreviations

SJS: Stevens-Johnson syndrome
TEN: Toxic epidermal necrolysis
PAS: Periodic acid-Schiff
MCP-1: Monocyte chemoattractant protein-1
IL: Interleukin
OSDI: Ocular surface disease index
FL: Fluorescent staining
TBUT: Tear break-up time
LIPCOF: Lid parallel conjunctival folds
PBS: Phosphate buffered saline
BSA: Bovine serum albumin
DAPI: 4',6-Diamino-2-phenylindole
GM-CSF: Granulocyte–macrophage colony stimulating factor
CXCL11: C-X-C motif chemokine ligand 11
CX3CL1: C-X3-C motif chemokine ligand 1
IFN-γ: Interferon gamma
TNF-α: Tumor necrosis factor alpha
CCL: C–C motif chemokine ligand
SD: Standard deviation
