# Supplementary material for: Ocular surface involvement and histopathologic changes in the acute stage of Stevens-Johnson syndrome and toxic epidermal necrolysis: a cross-sectional study
Source: BMC Ophthalmol. 2023 Jul 3;23:297. doi: 10.1186/s12886-023-03052-7 (PMC10316600; doi:10.1186/s12886-023-03052-7)
Supplement: Supplementary file 1 — Additional file 1: Fig. S1. The box plot of tear cytokines level between male vs female in acute SJS/TEN patients and healthy volunteers. *P value <0.05; **P value <0.01; ***P value <0.001; ns: not significant; GM-CSF: granulocyte-macrophage colony stimulating factor; CXCL11: C-X-C motif chemokine ligand 11; CX3CL1: C-X3-C motif chemokine ligand 1; IFN-γ: interferon gamma; TNF-ɑ: tumor necrosis factor alpha; IL: interleukin; CCL: C-C motif chemokine ligand. [file 12886_2023_3052_MOESM1_ESM.pptx]

## Slide 1
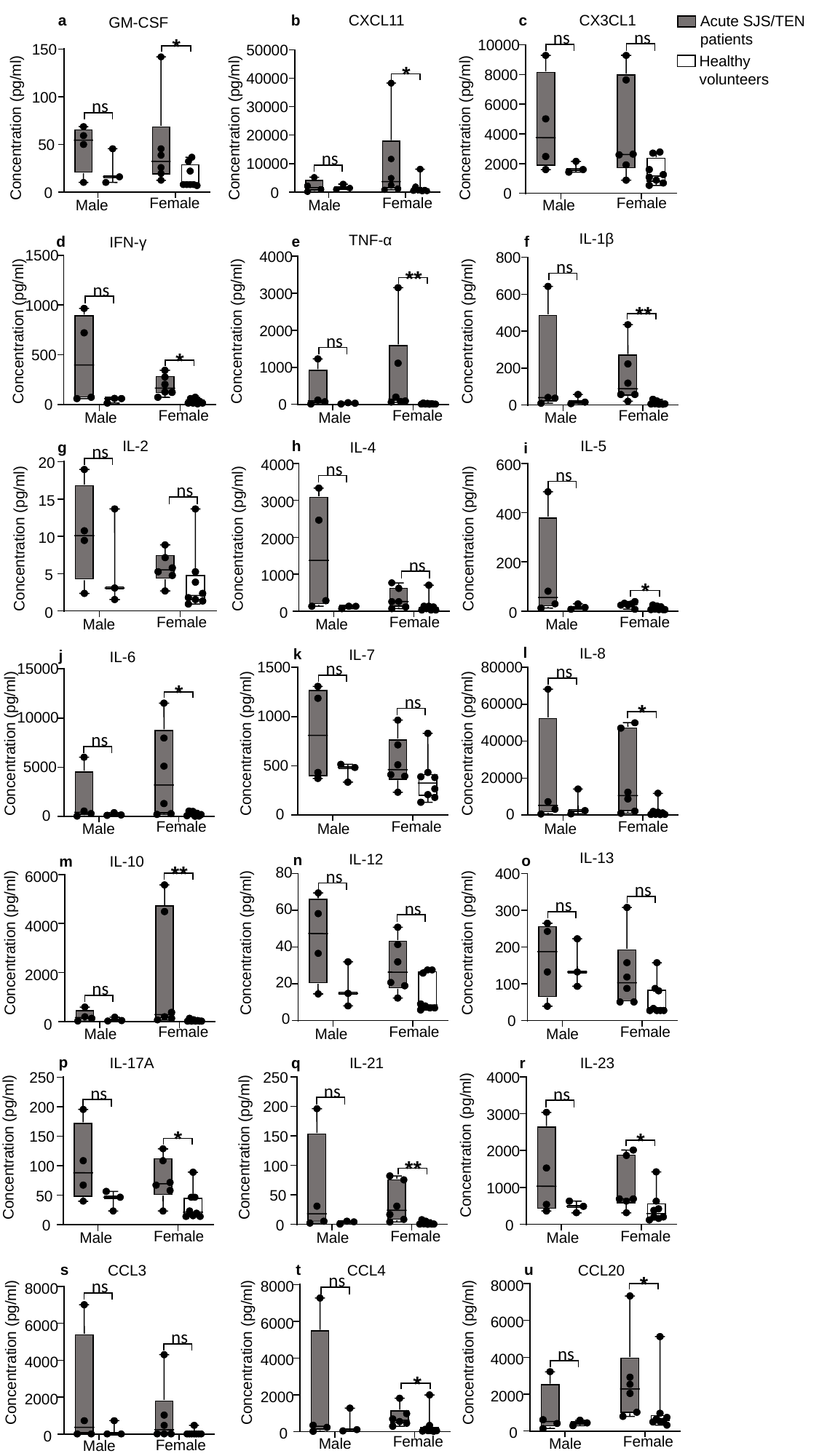

a
b
c
CXCL11
CX3CL1
GM-CSF
ns
ns
*
10000
150
50000
*
8000
40000
100
ns
6000
30000
Concentration (pg/ml)
Concentration (pg/ml)
Concentration (pg/ml)
4000
20000
50
ns
10000
2000
0
0
0
Female
Male
Female
Male
Female
Male
Acute SJS/TEN
patients
Healthy
volunteers
IL-1β
TNF-α
d
e
f
IFN-γ
1500
4000
800
ns
**
ns
3000
600
**
1000
2000
400
Concentration (pg/ml)
Concentration (pg/ml)
Concentration (pg/ml)
ns
*
500
1000
200
0
0
0
Female
Male
Female
Male
Female
Male
ns
IL-5
h
IL-2
g
IL-4
i
ns
20
4000
600
ns
ns
15
3000
400
10
2000
Concentration (pg/ml)
Concentration (pg/ml)
Concentration (pg/ml)
ns
200
5
1000
*
0
0
0
Female
Male
Female
Male
Female
Male
l
IL-8
k
IL-7
j
IL-6
ns
ns
1500
80000
15000
*
ns
*
60000
1000
10000
ns
40000
Concentration (pg/ml)
Concentration (pg/ml)
Concentration (pg/ml)
500
5000
20000
0
0
0
Female
Male
Female
Male
Female
Male
IL-13
IL-12
n
o
m
IL-10
**
ns
80
400
6000
ns
ns
ns
60
300
4000
Concentration (pg/ml)
Concentration (pg/ml)
Concentration (pg/ml)
40
200
2000
ns
20
100
0
0
0
Female
Male
Female
Male
Female
Male
p
IL-17A
IL-21
r
IL-23
q
250
4000
250
ns
ns
ns
200
200
3000
*
*
150
150
Concentration (pg/ml)
Concentration (pg/ml)
Concentration (pg/ml)
2000
**
100
100
1000
50
50
0
0
0
Female
Male
Female
Male
Female
Male
s
t
u
CCL3
CCL4
CCL20
ns
*
ns
8000
8000
8000
6000
6000
6000
ns
ns
Concentration (pg/ml)
Concentration (pg/ml)
Concentration (pg/ml)
4000
4000
4000
*
2000
2000
2000
0
0
0
Female
Male
Female
Male
Female
Male
